# Progress and challenges in exploring the pathogenesis of depressive disorders via RNA sequencing

**DOI:** 10.3389/fgene.2026.1792849

**Published:** 2026-08-28

**Authors:** Boyu Zhang, Shizhong Zheng, Lihua Guo, Hong Zhao

**Affiliations:** 1 Department of Acupuncture and Moxibustion, Shanghai University of Traditional Chinese Medicine, Shenzhen Hospital, Shenzhen, China; 2 Shanghai University of Traditional Chinese Medicine, Shanghai, China

**Keywords:** biomarkers, depressive disorder, molecular mechanisms, RNA-seq, transcriptomics

## Abstract

Depressive disorders (DD) are highly heterogeneous psychiatric illnesses. Their complex pathogenesis, coupled with suboptimal response rates to current clinical treatments, poses significant challenges to global mental health. High-throughput RNA sequencing (RNA-seq) has emerged as a powerful tool for dissecting the molecular mechanisms of DD at the transcriptomic level. This paper reviews recent advances utilizing bulk RNA-seq, single-cell RNA-seq (scRNA-seq), and single-nucleus RNA-seq (snRNA-seq) to explore the pathogenesis of major depressive disorder (MDD), perinatal depression (PND), perimenopausal depression (PMD), and post-stroke depression (PSD). Current evidence indicates that immune-inflammatory activation, impaired synaptic plasticity, and mitochondrial energy metabolism dysfunction constitute shared core pathological mechanisms across these disorders, validating classical pathogenic hypotheses at the molecular level. Furthermore, distinct clinical subtypes exhibit significant transcriptomic heterogeneity; for instance, MDD shows marked sexual dimorphism and immunophenotypic characteristics. Specific molecular pathways also distinguish different DD categories: PND and PMD mainly involve imbalances in neuroendocrine–immune interaction networks driven by hormonal fluctuations, whereas PSD is characterized by the interaction between innate immune responses and autophagy flux impairment. Although RNA-seq has significantly advanced our understanding of DD, challenges such as restricted sample sources, high data heterogeneity, and insufficient clinical translation remain. Future research should prioritize single-cell and spatial transcriptomics to resolve tissue heterogeneity, promote multi-omics integration to construct causal regulatory networks, and strengthen cross-species comparative studies. These efforts will accelerate the development of precision diagnosis and personalized treatment strategies.

## 1 Introduction

Depressive disorders (DD) are common affective mental disorders characterized by core symptoms including persistent low mood, reduced interest or anhedonia, often accompanied by psychomotor retardation, cognitive impairment, decreased activity levels, anxiety, alterations in sleep and eating behaviors, difficulty concentrating, and recurring suicidal thoughts (Clack and Ward, 2019). Statistics indicate that by the end of 2015, approximately 300 million people worldwide were suffering from depressive disorders, and the prevalence has significantly increased since the global COVID-19 pandemic (Herrman et al., 2019; Kupcova et al., 2023). It is projected that by 2030, depressive disorders will become the leading cause of the global disease burden, as predicted by the World Health Organization (Malhi and Mann, 2018). Therefore, the prevention and treatment of depressive disorders have attracted widespread attention from researchers.

**FIGURE 1:**
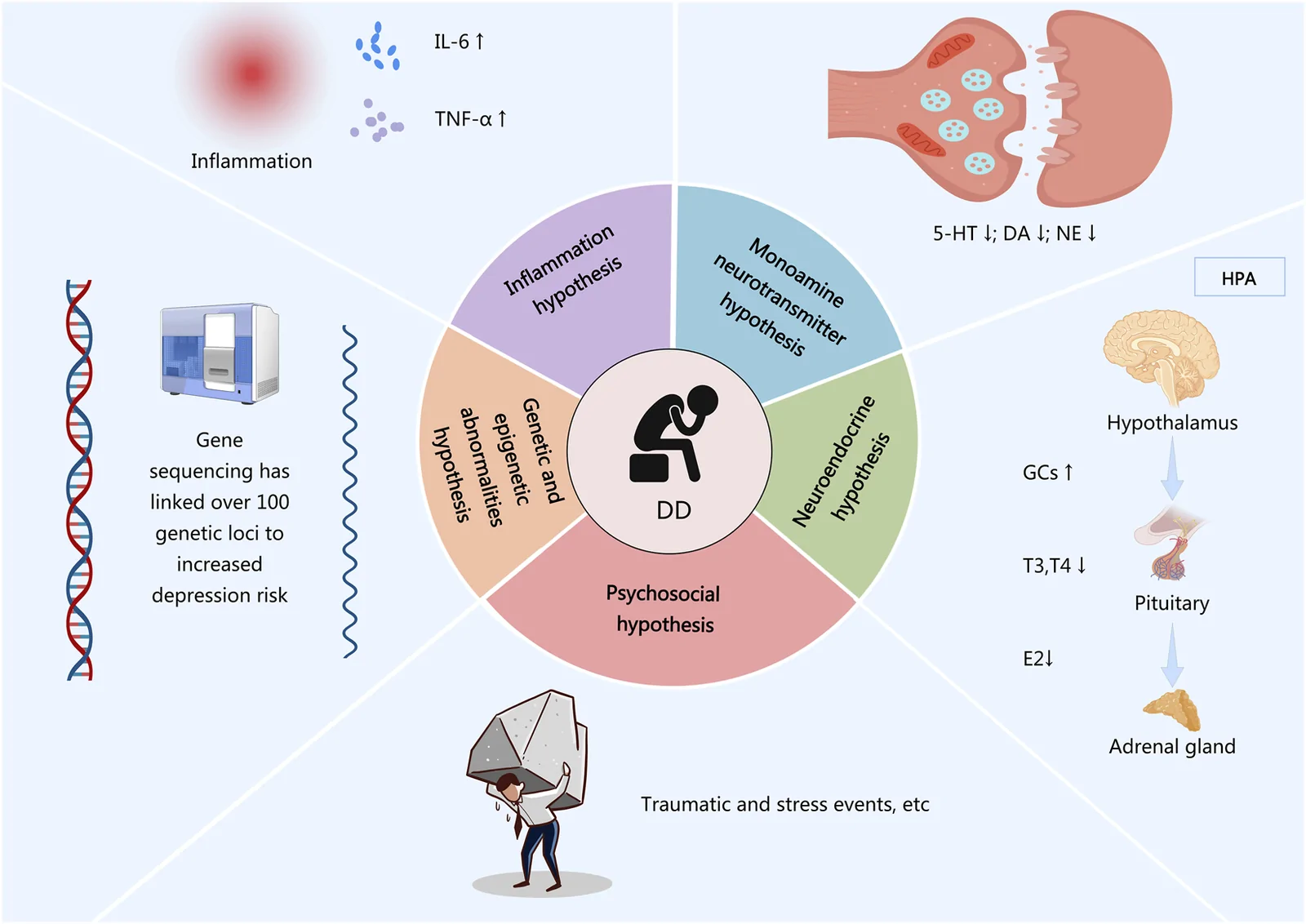
Schematic diagram of the hypothetical mechanism for the development of depressive disorders.

Depressive disorders result from the complex interplay of various biological, psychological, and socio-environmental factors, involving intricate underlying mechanisms. At present, the primary hypotheses explaining the etiology of depressive disorders include the monoamine hypothesis, the neuroendocrine hypothesis, the inflammatory hypothesis, the genetic and epigenetic anomalies hypothesis, and the psychosocial hypothesis,as shown in Figure 1. The classic monoamine hypothesis, first proposed and widely accepted, posits that depression is closely associated with a functional deficiency of monoamine neurotransmitters in the brain, such as serotonin (5-HT), norepinephrine (NE), and dopamine (DA) (Malhi and Mann, 2018). As research progresses, the neuroendocrine hypothesis has emphasized the imbalance of the hypothalamic-pituitary-adrenal (HPA) axis. Overactivation of the HPA axis leads to excessive secretion of glucocorticoids (such as cortisol), which in turn alters neurotransmitter metabolism by disrupting negative feedback regulation and impairing hippocampal neurogenesis (Pariante and Lightman, 2008). The inflammatory hypothesis posits that the activation of peripheral and central immune systems is a key pathologic aspect, characterized by elevated levels of pro-inflammatory cytokines (such as IL-6 and TNF-α), which can directly interfere with monoamine synthesis and impact neural circuitry function (Miller and Raison, 2016). At the genetic level, large-scale consortium efforts (such as the PsychENCODE Consortium) and Genome-Wide Association Studies (GWAS) have established a foundational molecular framework for depression. For instance, a large-scale meta-analysis identified over 100 genetic loci significantly associated with the risk of depression, revealing the genetic susceptibility of genes related to synaptic structures and neurotransmission (Howard et al., 2019). Integrating these genetic risk factors with transcriptomic data is crucial for interpreting the causal mechanisms of the disease. In addition, psychosocial factors are important precipitants, with long-term stress triggering a cascade of reactions at the physiological, neuroendocrine, and immune levels, ultimately promoting the manifestation of the depressive phenotype.

Although these classical hypotheses each have their focus, they are interconnected within a complex network, and a single mechanism is unlikely to fully explain the high heterogeneity of DD. Therefore, comprehensively understanding the pathogenesis of DD and identifying objective biological markers to devise new and more effective treatment strategies has become a core challenge and urgent need in this field (Stachowicz and Sowa-Kućma, 2022).

In the past decade, RNA-Seq technology has rapidly evolved, providing a powerful tool for analyzing disease characteristics and pathogenesis at the transcriptome level (Li et al., 2022; Xiong et al., 2021; Finotello and Di, 2015). Compared to traditional microarray technology, RNA-seq offers advantages such as high sensitivity, high throughput, high precision, and a broad detection range (Wang et al., 2009). In research related to DD, commonly used RNA sequencing technologies mainly include bulk RNA-seq, single-cell RNA-seq (scRNA-seq), and single-nucleus RNA sequencing (snRNA-seq). Bulk RNA-seq measures tissue or cell populations as units, obtaining population-averaged expression profiles suitable for identifying overall differences and pathway changes. It is widely used in the analysis of human post-mortem brain tissue, peripheral blood, and animal models (Conesa et al., 2016). The main limitation is that the heterogeneity of cellular composition can lead to bias, making it difficult to resolve cellular heterogeneity. Single-cell RNA sequencing (scRNA-seq) technology can comprehensively elucidate the complexity and heterogeneity of cells in different diseases (Papalexi and Satija, 2018; Hedlund and Deng, 2018). After isolating individual cells, whole-genome amplification and high-throughput sequencing are performed on each cell, thereby obtaining genetic information and gene expression profiles at the single-cell level (Ziegenhain et al., 2017). SnRNA-seq is capable of reliably acquiring nuclear transcriptional information under conditions with severe RNA degradation. It is applicable to tissues that are cryopreserved or difficult to dissociate (such as post-mortem human brain, myocardium, and fibrotic tissues), reducing dissociation stress and better recovering adherent or delicate cell types. Consequently, it offers complementary advantages over scRNA-seq in complex tissues such as the human brain (Denisenko et al., 2020).

Despite these technological advancements, the high clinical and genetic heterogeneity of depressive disorders implies that applying RNA-seq to study isolated subtypes provides a limited perspective on the overarching pathology. To address this gap and move beyond a mere catalog of individual studies, this review aims to construct a comparative transcriptomic framework across distinct clinical entities. We selected major depressive disorder (MDD) as the classical idiopathic type; perinatal (PND) and perimenopausal depression (PMD) as subtype driven by reproductive hormonal transitions; and post-stroke depression (PSD) as a subtype triggered by acute vascular injury. By systematically comparing bulk, scRNA-seq, and snRNA-seq transcriptomic evidence across these domains, our primary objective is to explicitly evaluate shared versus distinct molecular mechanisms. We seek to disentangle the core pathological signatures common to depressive phenotypes from etiology-specific pathways. Ultimately, this comparative synthesis aims to provide a unified molecular perspective to guide precision diagnosis and subtype-specific therapeutic strategies.

## 2 Major depressive disorder

Major depressive disorder (MDD) is one of the most common types of depressive disorders. Studies have reported that following the COVID-19 pandemic in 2020, the estimated global prevalence of MDD was 3,152.9 cases per 100,000 (95% UI 2,722.5-3,654.5), affecting approximately 246 million people (COVID-19 Mental Disorders Collaborators 2021). MDD may persist throughout the entire life course and is associated with an increased incidence of diabetes, cardiovascular disease, stroke, and even suicide (World Health Organization, 2017; Krittanawong et al., 2023; Liu et al., 2025). Suicidal ideation (SI) is an early warning signal for potential suicidal behavior, commonly seen in MDD patients, and represents a key target for timely intervention (Klonsky et al., 2021; Cai et al., 2021). However, there is still a lack of molecular biomarkers for the treatment of MDD and SI (Ling et al., 2024).

In recent years, transcriptomic studies based on human post-mortem brain tissue and peripheral blood have provided important clues for elucidating the core molecular mechanisms of MDD, sex differences, and the molecular heterogeneity of clinical subtypes. The related studies, along with the main differentially expressed genes (DEGs) and biological processes identified, are summarized in Table 1.

**TABLE 1:** Transcriptomic studies related to major depressive disorder (MDD).

Author (Year) (Ref)	Sample type	Technology	Sample size (N)	Medication and specific drug class	Key hub DEGs and effect size	Enriched pathways/Biological processes	Independent cohort validation
Yoshino et al. (2021)	Post-mortem human dlPFC (BA46)	Bulk RNA-seq	15 MDD vs. 15 HC	MDD: 9/15 drug-free; 6/15 positive for Antidepressants (specific class unspecified) HC: 100% drug-free	Up: ZFP36 (FC = 2.18), ELK1 (FC = 1.49), TLR2 (FC > 1.3) Down: HES5 (FC = 0.54), ASPDH (FC = 0.77),RBBP4 (FC = 0.75)	Immune and inflammatory response, transcriptional regulation	No
Pantazatos et al. (2017)	Post-mortem human dlPFC (BA9)	Bulk RNA-seq	9 MDD_nSI vs. 21 MDD_SI vs. 29 HC	MDD and HC: 100% drug-free	Up: MTRNR2L8 (log2FC = 0.67) Down:SERPINH1 (log2FC = −0.72), IL8 (log2FC = −0.53), CCL4(log2FC = −0.59)	Cytokine signaling, immune response, apoptosis regulation	No
Mahajan e al. (2018)	Post-mortem human hippocampal dentate gyrus	Bulk RNA-seq	23 MDD vs. 23 HC	MDD: 22/23 drug-free; 1/23 positive for TCA and Benzodiazepine HC: 100% drug-free	Up: CCL2 (log2FC = 1.70), ADM ((log2FC = 1.11), ADAMTS9 ((log2FC = 1.36), KANSL1 ((log2FC = 1.12) Down: ISG15 (log2FC = −1.08), IFI44L ((log2FC = −0.93), IFI6((log2FC = −0.92), NR4A1 ((log2FC = −0.95)	Interferon signaling, immune/inflammatory response, angiogenesis	No
Labonté et al. (2017)	Post-mortem human multi-region brain; Mouse brain	Bulk RNA-seq	Human: 26 MDD vs. 22 HC; Mouse: 20 CVS vs. 20 SR	MDD: Mixed (specific drug classes unspecified) HC: 100% drug-free	Female Down: DUSP6(FC unspecified) Male Up: EMX1 (FC unspecified)	ERK/MAPK signaling cascade, neurogenesis	Yes
Maitra et al. (2023)	Post-mortem human dlPFC (BA9) nuclei	snRNA-seq	Female: 20 MDD vs. 18 HC; Male: 17 MDD vs. 16 HC	MDD: Mixed (specific class unspecified) HC: 100% drug-free	Female Up:ROBO2, ADAMTSL1, SLIT3, THSD4(FC unspecified) Female Down:SOCS3(FC unspecified) Male: Specific hub DEGs not detailed in main text (FC unspecified)	Microglia-neuron interaction, axon guidance (ROBO/SLIT), extracellular matrix organization	No
Isslet et al. (2020)	Post-mortem human multi-region brain (GSE102556)	Bulk RNA-seq	26 MDD vs. 22 HC	MDD: Mixed (specific class unspecified) HC: 100% drug-free	Female Down: LINC00473 (FC > 1.3) Male: Not significantly regulated	CREB/cAMP signaling, synaptic plasticity, stress response	Yes
Chen et al. (2025)	Post-mortem human dlPFC (BA9) nuclei; Human peripheral blood (GSE144136, GSE38206)	snRNA-seq; Bulk RNA-seq	snRNA: 17 MDD vs. 16 HC; Bulk: 9 MDD vs. 9 HC	Unspecified	Up:CSTB(FC unspecified) Down:CASKIN1 (FC unspecified)	Synaptic plasticity, vesicle trafficking	No
Xu et al. (2024)	Post-mortem human dlPFC (BA9) (GSE101521)	Bulk RNA-seq	6 MDD_nSI vs. 13 MDD_SI vs. 23 HC	Unspecified	Up:HMGB1,STAT3,p65 (FC unspecified)	Microglial activation, autophagy, NF-κB/STAT3 inflammatory signaling	Yes
Wang et al. (2022)	Human peripheral blood	Bulk RNA-seq	15 MDD vs. 15 HC	MDD and HC: 100% drug-free	Up: RPS11 (log2FC = 4.07), CFL1 (log2FC = 2.40), MALAT1 (log2FC = 0.69) Down: ATP2A3 (log2FC = −1.91)	Energy metabolism, inflammation, translation/ribosome	No
Sforzini et al. (2025)	Human peripheral blood	Bulk RNA-seq	105 MDD vs. 34 HC	MDD: 22/106 drug-free; 84/106 positive for Antidepressants (specific class unspecified) HC: 100% drug-free	Up:TNFSF9, TNFSF10, TNFSF15,MMP8, MMP19, MMP25, MMP28 (FC unspecified)	Immunometabolic pathways, inflammation, cell-cycle, extracellular matrix remodeling	No
Wang M. et al. (2025)	Human peripheral blood	Bulk RNA-seq	75 MDD_SI vs. 82 MDD_nSI vs. 149 HC	MDD and HC: 100% drug-free	Up: SYK, STAT3, IL1B,TP53,EP300 (FC unspecified) Down: EIF4A3, SRSF11, SF3B1,FAU,RPS3 (FC unspecified)	RNA splicing Immunity/Inflammation, Energy metabolism	No
Sirés et al. (2025)	Human peripheral blood	Bulk RNA-seq	150 TRD vs. 143 non-TRD	293/293 positive for Antidepressants (SSRIs, SNRIs, TCAs, NaSSAs, etc.)	Up: NTNG1 (log2FC = 0.66), PTPN20 (log2FC = 0.54) Down: DAAM2 (log2FC = −0.79), PHF24 (log2FC = −0.66), FOSB (log2FC = −0.57)	Innate immune response, ncRNA processing, epigenetic regulation	No
Hofstra et al. (2024)	Mouse hippocampus; Human post-mortem hippocampus (GSE53987)	Bulk RNA-seq	Mouse: 9 MS vs. 12 SR; Human: 17 MDD vs. 18 HC	Unspecified	Mouse Up: Gng5 (log2FC = 0.51), Ndufa2 (log2FC = 0.52), Tomm7 (log2FC = 0.49) Mouse Down: Zfp46 (log2FC = −0.38), Eps8l1 (log2FC = −0.40) Human Down: GNG5, ATP5MG, COX5A, COX6B1 (FC unspecified)	Mitochondrial stress response, oxidative phosphorylation, protein folding	Yes

MDD, Major Depressive Disorder; HC, Healthy Control; TRD/non-TRD, Treatment-Resistant/non-Treatment-Resistant Depression; SI/nSI, Suicidal Ideation/non-Suicidal Ideation; dlPFC, Dorsolateral Prefrontal Cortex; BA, Brodmann Area; snRNA-seq, Single-nucleus RNA sequencing; CVS, Chronic Variable Stress; SR, Standard Rearing; MS, Maternal Separation; TCA/SSRIs/SNRIs/NaSSAs, Antidepressant classes; DEGs, Differentially Expressed Genes; NA, not accepted; FC, Fold Change. Medication and Specific Drug Class: Drug-free (no recent antidepressant use, typically toxicology-confirmed); Mixed (comprising both medicated and unmedicated subjects); Unspecified (not explicitly reported).

### 2.1 Transcriptomic studies of brain tissue post-mortem

Initial transcriptomic studies of post-mortem brain tissue primarily utilized bulk RNA-seq, yet integrating these data requires careful consideration of tissue heterogeneity. For instance, Yoshino et al. (2021) sequenced purified synaptosomes from the dlPFC, revealing large-scale gene expression shifts enriched in immune response and apoptotic pathways. These findings specifically link synaptic-level neuroinflammation to MDD-related plasticity deficits. In contrast, Pantazatos et al. (2017) analyzed whole-tissue homogenates, uncovering a broader pathological landscape characterized by microglial and endothelial suppression alongside glial dysfunction. Notably, their high-resolution exon-level analysis identified alternative splicing in ATP9B—a gene critical for vesicle transport—underscoring the value of incorporating post-transcriptional modifications into multi-omic frameworks. Beyond analytical pipelines, neuroanatomical site selection profoundly impacts outcomes, as the dlPFC and hippocampus possess distinct transcriptomic architectures. Consequently, regional findings—such as the localized impaired angiogenesis and aberrant interferon signaling identified in the hippocampal dentate gyrus (Mahajan et al., 2018)—often reflect intrinsic brain compartmentalization rather than conflicting evidence. Ultimately, discrepancies across the literature are frequently methodological products of differing tissue fractionation methods and analytical workflows rather than true biological contradictions.

The sample sizes in these early research cohorts were relatively small (e.g., N = 15 to 29 per group) (Yoshino et al., 2021; Pantazatos et al., 2017; Mahajan et al., 2018), which limited the statistical power and increased the risk of differences in the results. Subsequent research utilizing larger cohorts has revealed that MDD transcriptome remodeling is highly sex-specific. A multi-regional RNA-seq study demonstrated that only 5%–10% of differentially expressed genes overlap between sexes, with female profiles enriched in mitogen-activated protein kinase (MAPK) signaling and male profiles in synaptic transmission (Labonté et al., 2017). This sexual dimorphism extends to non-coding RNAs, such as the female-specific downregulation of the lncRNA LINC004730 (Issler et al., 2020). The significant sex-specific divergence suggests that combining male and female samples in bulk analyses may obscure key pathological signals (Labonté et al., 2017; Issler et al., 2020).

To address the limitations of bulk sequencing and resolve cellular heterogeneity, recent studies have applied single-nucleus RNA sequencing (snRNA-seq). By profiling isolated nuclei, snRNA-seq helps differentiate cell-state alterations from compositional changes. For example, a snRNA-seq analysis of the dlPFC confirmed sex-specific differences at the cellular level, revealing that female DEGs are predominantly enriched in microglia and parvalbumin interneurons, whereas male DEGs are concentrated in deep-layer excitatory neurons, astrocytes, and oligodendrocytes (Maitra et al., 2023). Further snRNA-seq studies have validated these findings, identifying male-specific synaptic plasticity impairments in excitatory neurons driven by the miR-21-5p-CASKIN1 axis (Chen et al., 2025).

In summary, evidence from post-mortem brain tissues indicates that MDD pathology centers on neuroinflammation, synaptic impairment, and glial dysfunction. However, future interpretations must account for technological limitations and demographic variables. Findings from bulk RNA-seq should be corroborated by single-cell resolution data to explicitly distinguish true cell-state shifts from reactive cell-composition changes, and sex-stratification should be adopted as a standard analytical practice.

### 2.2 Transcriptomic studies of human peripheral blood

Although transcriptomic profiling of post-mortem brain tissues provides direct evidence of neural dysfunction, the severe scarcity and restricted availability of these samples preclude their application in identifying dynamic biomarkers. Consequently, the peripheral blood transcriptome has emerged as a critical systemic surrogate for MDD pathology. Recent bulk RNA-seq profiling has consistently identified systemic immune-inflammatory activation and perturbed cellular bioenergetics—particularly oxidative phosphorylation and chemokine signaling—as core peripheral signatures, largely mirroring the transcriptomic landscape of the central nervous system (Xu et al., 2024; Wang Y. et al., 2022).

Nevertheless, translating these peripheral signatures into clinical utility requires navigating the profound confounding effects of psychotropic medications. To establish a rigorous hierarchy of evidence, it is essential to contextualize the pharmacological baseline of each cohort, as different drug classes—such as selective serotonin reuptake inhibitors (SSRIs), serotonin-norepinephrine reuptake inhibitors (SNRIs), and tricyclic antidepressants (TCAs)—exert distinct transcriptomic reprogramming effects.

Studies utilizing strictly drug-free cohorts provide the most unambiguous baseline for identifying intrinsic disease pathology. For example, RNA-seq analyses of entirely unmedicated cohorts have reliably confirmed that widespread immune-inflammatory activation and energy metabolism abnormalities are core baseline signatures of MDD (Wang Y. et al., 2022). Furthermore, analyzing drug-free cohorts has successfully delineated distinct modules for specific clinical phenotypes; for instance, suicidal ideation (MDD-SI) specific modules are predominantly driven by RNA splicing and immune-inflammatory responses, whereas non-SI depression (MDD-nSI) is closely related to innate immunity, mitochondrial function, and neurodegenerative pathways (Wang M. et al., 2025).

Conversely, transcriptomic profiles derived from medicated cohorts—even when medication status is appropriately adjusted as a statistical covariate—warrant nuanced interpretation. Moving beyond MDD as a monolithic entity, patient stratification based on high-sensitivity C-reactive protein (CRP) levels revealed that patients with elevated CRP (≥1 mg/L) exhibit widespread activation of immune-metabolic pathways (encompassing innate immunity and defense responses), whereas those with lower CRP (<1 mg/L) are primarily characterized by the suppression of cell cycle-related pathways (Sforzini et al., 2025). However, given that a substantial majority of this cohort (84 of 106) were receiving active pharmacotherapy, these profiles must be critically interpreted within their specific pharmacological context (Sforzini et al., 2025).

Similarly, peripheral RNA-seq has proven instrumental in conceptualizing treatment-resistant depression (TRD) as an independent molecular biotype characterized by pervasive epigenetic dysregulation and suppressed adaptive and innate immunity, rather than classical monoaminergic deficits (Sirés et al., 2025). Crucially, this TRD cohort was actively receiving a diverse array of antidepressants (including SSRIs, SNRIs, TCAs, and NaSSAs) (Sirés et al., 2025). While biostatistical models can partially regress the main effects of these drugs, prolonged exposure inherently alters the systemic immune landscape. Therefore, the immune suppression observed in TRD—which diverges significantly from the immune activation typically seen in drug-free cohorts (Wang Y. et al., 2022; Wang M. et al., 2025)—may reflect not only a distinct pathological biotype but also the complex immunomodulatory effects of sustained antidepressant therapy.

Collectively, while peripheral blood robustly captures the systemic immunometabolic reprogramming intrinsic to MDD, extracting true pathological signatures requires future studies to explicitly stratify data by distinct drug classes. This approach will help more accurately disentangle drug-induced transcriptomic shifts from the intrinsic pathophysiology of MDD.

### 2.3 Transcriptome studies of animal brain tissue

While human post-mortem studies are indispensable for identifying terminal pathological signatures, they are inherently cross-sectional and frequently confounded by lifelong environmental exposures. To elucidate the causal trajectories of specific pathogenic triggers—such as early life stress (ELS)—animal models remain strictly required. However, a major methodological challenge in utilizing rodent models is their limited ability to fully phenocopy the complex transcriptomic architecture of the human cortex.

To navigate this translational barrier, recent investigations have increasingly adopted cross-species transcriptomic approaches to isolate evolutionarily conserved disease mechanisms. A study compared hippocampal tissue transcriptome data between mice with early life stress (ELS) and patients with MDD. The results revealed that ELS induces significant transcriptional changes in the hippocampus of mice, with Gene Ontology (GO) and Kyoto Encyclopedia of Genes and Genomes (KEGG) analyses showing that DEGs are primarily enriched in mitochondrial stress response and protein folding pathways. When the mouse DEGs were converted to their human orthologous genes and overlapped with the transcriptome data of MDD patients, six genes were identified with differential expression in both mice and humans: GNG5, TRIP6, ATP5MG, COX5A, COX6B1, and UQCRH. Four of these genes (ATP5MG, COX5A, COX6B1, and UQCRH) are directly involved in mitochondrial function, suggesting that mitochondrial stress responses in the hippocampus may be a common underlying mechanism between mice with ELS and human depression (Hofstra et al., 2024).

## 3 Perinatal depression

Perinatal Depression (PND), also known as prenatal and postnatal depression, encompasses prenatal depression and postpartum depression (PPD), and is one of the common psychiatric disorders during the perinatal period and puerperium (中华医学会妇产科学分会产科学组: 围产期抑郁症筛查与诊治专家共识, 2021). According to the Diagnostic and Statistical Manual of Mental Disorders, Fifth Edition (DSM-5) criteria, peripartum depression is not a separate diagnostic category but rather encompasses depressive disorders occurring during pregnancy or within 4 weeks postpartum, collectively referred to as MDD with peripartum onset; alternatively, if they do not meet the diagnostic criteria for MDD, they can be classified under “Other Specified Depressive Disorders” (Woody et al., 2017; Association, 2013). Perinatal depression may lead to hyperemesis gravidarum, preeclampsia, miscarriage, or preterm birth. In severe cases, it can result in self-harm, suicide, infanticide, or child abuse. Its potential adverse effects on the fetus or newborn include restricted fetal growth and development, low birth weight, and newborn feeding difficulties. Furthermore, children of affected mothers are more likely to exhibit emotional, behavioral, and psychological issues in childhood (Slomian et al., 2019; Farías-An et al., 2018; Wisner et al., 2013; Giallo et al., 2015; Lutkiewicz et al., 2020). t present, the biological mechanisms underlying peripartum depression are not yet clear (Payne and Maguire, 2019). Multiple transcriptome studies based on human peripheral blood, placenta, and animal models have provided important clues for elucidating molecular abnormalities in immune inflammation, energy metabolism, and neuroplasticity. The associated research and their main DEGs and biological processes are shown in Table 2.

**TABLE 2:** Transcriptomic studies related to perinatal depression (PND).

Author (Year) (Ref)	Sample type	Technology	Sample size (N)	Medication and specific drug class	Key hub DEGs and effect size	Enriched pathways/Biological processes	Independent cohort validation
Pan et al. (2018)	Human peripheral blood	Bulk RNA-seq	8 PPD vs. 10 HC	PPD&HC: 100% drug-free	Up: CXCL2 (log2FC = 0.85), IL1B (log2FC = 0.72), DUSP1 (log2FC = 0.45), INSR (log2FC = 0.43) Down: ADRB3 (log2FC = −2.88), IL13 (log2FC = −1.64), CNR1 (log2FC = −0.50), PPARG (log2FC = −0.46)	Appetite regulation, energy metabolism (oxidative phosphorylation, TCA cycle), immune/chemokine signaling	Yes
Mehta et al. (2021)	Human peripheral blood	Bulk RNA-seq	15 PPD vs. 122 HC	MDD/HC: 24/137 positive for current medication (specific class unspecified)	Up: TNFRSF17 (logFC = 0.38), JCHAIN (logFC = 0.28) Down: MMP8 (logFC = −0.51), OLFM4 (logFC = −0.05)	Immune response, neutrophil degranulation, innate immunity, estrogen signaling	Yes
Björvang et al. (2025)	Human peripheral blood	Bulk RNA-seq	HC vs. APD vs. PPD	Unspecified	Up: TNFRSF17,MMP8,ISG15, RSAD2 (FC unspecified)	Immune response and cell motility	No
Martinez et al. (2022)	Human term placental villus tissue	Bulk RNA-seq	11 Pregnant women (BAI ≥ 10 and EPDS ≥ 10) vs. 11 Controls	Unspecified	Down: CD8B (FC = −20.25), CD46 (FC = −5.13), IL7R (FC = −4.82), CCR4 (FC = −4.33), CD8A (FC = −4.23), CD151 (FC = −3.65)	Primary immunodeficiency, T cell-mediated immunity, interleukin/cytokine signaling	No
Fu et al. (2025)	Mouse hippocampus and prefrontal cortex	Bulk RNA-seq	3 Sham vs. 3 HSP vs. 3 HSP + music	NA	Hip-Up: Sele (FC unspecified) Hip-Down: Wnt6, Sfrp5, Nupr1 (FC unspecified) mPFC-Up: Eya4 (FC unspecified) mPFC-Down: Lhx8 (FC unspecified)	Synaptic plasticity, Wnt signaling, oxidative stress, neuroinflammation	No
Ye et al. (2023)	Rat hippocampus	Bulk RNA-seq	10 Sham vs. 10 HSP vs. 10 HSPP	NA	Up: Sfrp1 (FC unspecified) Down: β-catenin, Dkk2, Fzd5 (FC unspecified)	Wnt signaling, Hippo signaling, amino acid metabolism,GABAergic/glutamatergic synaptic plasticity	No
Zhu and Tang (2020)	Mouse hippocampus	Bulk RNA-seq	Sham vs. HSP	NA	Up: Gm14205 (FC unspecified), NLRP3 (FC unspecified) Down: OXTR (FC unspecified)	OXTR (Oxytocin receptor) signaling, NLRP3 inflammasome activation, astrocytic neuroinflammation	No

PND, Perinatal Depression; PPD, Postpartum Depression; APD, Antenatal Depression; HC, Healthy Control; MDD, Major Depressive Disorder; EPDS, Edinburgh Postnatal Depression Scale; BAI, Beck Anxiety Inventory; Hip, Hippocampus; mPFC, Medial Prefrontal Cortex; HSP, Hormone-simulated pseudopregnancy; HSPP, Hormones-simulated postpartum period; RNA-seq, RNA sequencing; DEGs, Differentially Expressed Genes; NA, not accepted; FC, Fold Change. Medication and Specific Drug Class: Drug-free (no recent antidepressant use, typically toxicology-confirmed); Mixed (comprising both medicated and unmedicated subjects); Unspecified (not explicitly reported).

### 3.1 Transcriptomic studies of human peripheral blood

Providing the highest tier of evidence for intrinsic PND pathology, RNA-seq of peripheral blood mononuclear cells (PBMCs) from a strictly drug-free cohort of postpartum depression (PPD) patients revealed a robust upregulation of energy metabolism and immune response pathways, alongside a marked suppression of DNA mismatch repair mechanisms (Pan et al., 2018). Notably, the differential expression of polygenes associated with appetite regulation and nutrient response underscores the specific significance of energy intake and weight regulation networks in the drug-naive perinatal depressive phenotype (Pan et al., 2018).

Furthermore, longitudinal whole-blood RNA-seq studies spanning late pregnancy to the puerperal period indicate that transcriptomic alterations occur prior to the clinical onset of symptoms (Björvang et al., 2025; Martinez et al., 2022; Fu et al., 2025; Ye et al., 2023). Specifically, dysregulated immune and inflammatory signatures (including key candidate genes such as TNFRSF17, MMP8, ISG15, and RSAD2) observed during the third trimester are consistently associated with the subsequent manifestation of PPD (Mehta et al., 2021; Petralia et al., 2019; Song et al., 2013; Björvang et al., 2025). However, it is important to critically note that several of these longitudinal cohorts (e.g., (Mehta et al., 2021)) included patients actively receiving psychotropic medications. While these studies robustly confirm that immune-related molecular characteristics are deeply embedded within PND, interpreting the exact magnitude of these transcriptomic shifts must carefully account for potential pharmacological confounds.

### 3.2 Transcriptomic studies of human placental tissues

Expanding beyond maternal circulation, PND pathophysiology significantly impacts the maternal-fetal interface, potentially mediating cross-generational effects through “early life programming”. Transcriptomic analysis of term placental tissues from clinically matched cohorts demonstrated a pronounced suppression of immune-related networks in mothers experiencing significant prenatal anxiety and depression (Martinez et al., 2022). Specifically, the widespread downregulation of key immunoregulatory transcripts—spanning T-cell regulation, interleukin signaling, and innate immunity (e.g., CD46, CD15, CD8α/β, IL7Rα, and CCR4)—suggests that maternal affective dysregulation compromises the immune defense capacity of the placental barrier, thereby perturbing the fetal intrauterine developmental trajectory (Martinez et al., 2022).

### 3.3 Transcriptomic studies of animal brain tissues

Mechanistic insights into the central nervous system alterations driven by peripartum hormonal fluctuations have been extensively derived from the hormone-simulated pregnancy (HSP) rodent model. A hippocampal and prefrontal RNA-seq analyses reveal a dual pathogenic cascade: the suppression of synaptic plasticity networks coupled with the generalized hyperactivation of neuroinflammatory and oxidative stress pathways (Fu et al., 2025).

At the synaptic level, transcriptomic evaluations indicate that deficient canonical Wnt/β-catenin signaling disrupts the excitatory/inhibitory neurotransmission balance in hippocampal CA1 pyramidal neurons; crucially, pharmacological activation of the Wnt pathway effectively restores this balance and alleviates depressive-like behaviors such as anhedonia (Ye et al., 2023). Concurrently, neuroinflammation is tightly orchestrated by specific non-coding RNAs. In HSP-induced PPD models, the upregulation of lncRNA Gm14205 directly targets and suppresses the oxytocin receptor (OXTR), thereby unleashing astrocytic NLRP3 inflammasome activation and subsequent IL-1β secretion (Zhu and Tang, 2020). Together, these findings delineate a hormone withdrawal-triggered axis—encompassing Wnt-mediated synaptic impairment and lncRNA-driven glial inflammation—as a core central mechanism of PPD.

## 4 Perimenopausal depression

Perimenopause is the transitional phase for women from their reproductive to non-reproductive years, typically occurring between the ages of 40 and 55. During this period, there is a significant fluctuation in hormone levels in the body, which can easily lead to a series of physiological and psychological changes (Brinton et al., 2015; Wang L. et al., 2025). According to the latest reports on women’s health from international mental health research, the likelihood of perimenopausal or postmenopausal women developing depression is three times that of premenopausal or menstruating women (Colvin et al., 2017). Perimenopausal depression (PMD) is a prominent public health issue, severely affecting the quality of life and sleep of middle-aged women and leading to a series of health problems including cognitive decline, cardiovascular diseases, osteoporosis, and sexual dysfunction (Tandon et al., 2022; Nazarpour et al., 2021; Maki et al., 2019). The corresponding RNA-seq studies and their main DEGs and biological processes are shown in Table 3.

**TABLE 3:** Transcriptomic studies related to perimenopausal depression (PMD).

Author (Year) (Ref)	Sample type	Technology	Sample size (N)	Medication and specific drug class	Key hub DEGs and effect size	Enriched pathways/Biological processes	Independent cohort validation
Feng et al. (2025)	Human PBMCs	Bulk RNA-seq	5 Pre-treat PMD vs. 5 Post-treat PMD	Pre-treat PMD: 100% drug-free	Up: FOSL1, OSM, CXCL8, IL1B (FC unspecified) Down: LINC01311 (FC unspecified)	Inflammatory/immune response, NF-κB signaling, IL-17 signaling, estrogen signaling pathway	No
Rudzinskas et al. (2021)	Human peripheral blood lymphoblastoid cell lines (LCLs)	Bulk RNA-seq	8 PMD vs. 9 HC	NA	Up: IGLL5 (FC = 28.14), CCL17 (FC = 5.23), UGT2B17 (FC = 3.69), LAPTM4B (FC = 3.06) Down: DEXI (FC = −2.39), MAP4K1 (FC = −1.95)	Steroid metabolism, pro-inflammatory immune response	No
Wang W. et al. (2022)	Mouse hypothalamus	Bulk RNA-seq	3 OVX vs. 3 Sham	NA	Up:Trpm2, Trpm8, Agrp, Pomc, Timeless, Gnrh, Avp, Lepr, Insr (FC unspecified)	Thermoregulation (Hot flush mechanisms), food intake/obesity pathways, neuroendocrine signaling	No

Main DEGs: PMD, Perimenopausal Depression; HC, Healthy Control; PBMCs, Peripheral Blood Mononuclear Cells; LCLs, Lymphoblastoid Cell Lines; OVX, Ovariectomy; RT-qPCR, Quantitative Reverse Transcription Polymerase Chain Reaction; lncRNA, Long non-coding RNA; TRP, Transient Receptor Potential; DEGs, Differentially Expressed Genes; NA, not accepted; FC, Fold Change. Medication and Specific Drug Class: Drug-free (no recent antidepressant use, typically toxicology-confirmed); Mixed (comprising both medicated and unmedicated subjects); Unspecified (not explicitly reported).

### 4.1 Transcriptomic studies of human peripheral blood

Providing a high tier of evidence strictly controlled for psychotropic confounds, peripheral blood transcriptomics highlight the intersection of immune hyperactivation and steroid metabolism as a hallmark of perimenopausal depression (PMD) (Feng et al., 2025; Rudzinskas et al., 2021). Profiling of PBMCs from a strictly drug-free baseline cohort of PMD patients revealed a marked overexpression of pro-inflammatory mediators (e.g., CXCL8, IL1B, FOSL1, OSM), characterized by enriched IL-6 and TNF-related signaling (Feng et al., 2025). Notably, longitudinal RNA-seq following a targeted traditional Chinese medicine intervention (Bushen Shugan Huayu decoction) demonstrated that the clinical alleviation of depressive symptoms correlated directly with the transcriptomic downregulation of these inflammatory nodes and the concurrent upregulation of putative neuroprotective non-coding RNAs (e.g., LINC01311) (Feng et al., 2025).

Furthermore, to definitively isolate the specific transcriptomic effects of estrogen withdrawal from any *in vivo* pharmacological or systemic confounds, *in vitro* models utilizing transformed lymphoblastoid cell lines (LCLs) from PMD patients identified persistent dysregulation in the inflammation-steroid metabolism network (Rudzinskas et al., 2021). Across baseline, estradiol exposure, and withdrawal conditions, LCLs exhibited continuous upregulation of the steroidogenic enzyme CYP7B1 and exacerbated expression of the pro-inflammatory chemokine CXCL10 upon estrogen withdrawal, establishing a highly controlled peripheral molecular vulnerability basis for PMD (Rudzinskas et al., 2021).

### 4.2 Transcriptome studies of animal brain tissues

An RNA-seq study compared the hypothalamic transcriptome between ovariectomized (OVX) and sham-operated mice, identifying 7,295 upregulated genes and 8,979 downregulated genes. GO and KEGG analyses revealed significant enrichment of these genes in thermoregulation, feeding and energy metabolism, glucose and lipid metabolism, cardiovascular regulation, circadian rhythms, and multiple endocrine and neurotransmitter-related signaling pathways, such as TRP channels, insulin, GnRH, and thyroid hormone pathways. Further validation showed that key genes related to hot flashes (including TRPM2 and TRPM8) and obesity (including Agrp, Pomc, Lepr, and Insr), as well as genes related to rhythm and homeostasis (including Timeless, Gnrh, and Avp), were abnormally expressed in the hypothalamus of OVX mice. This is consistent with the symptoms of menopausal depression, such as increased tail skin temperature, weight gain, and increased abdominal fat. These results suggest that estrogen deficiency can reshape the thermal, metabolic, and endocrine networks through multiple hypothalamic pathways, potentially participating in the occurrence of menopausal depression and related symptoms (Wang W. et al., 2022).

## 5 Post-stroke depression

Post-stroke depression (PSD) is a common sequel of stroke, affecting nearly one-third of stroke survivors. It severely impacts the functional prognosis of stroke patients and is associated with a high mortality rate (Robinson and Jorge, 2016; Gomberg et al., 2024). According to DSM-5 criteria, PSD can be formally diagnosed as a depressive disorder due to another medical condition (cerebrovascular stroke). PSD is characterized by persistent anhedonia and despair, and can lead to adverse outcomes, including functional impairment, reduced quality of life, recurrent stroke, and even death (Ferrari et al., 2024). Relevant RNA-seq studies and their main DEGs and biological processes are shown in Table 4.

**TABLE 4:** Transcriptomic studies related to post-stroke depression (PSD).

Author (Year) (Ref)	Sample type	Technology	Sample size (N)	Medication and specific drug class	Key hub DEGs and effect size	Enriched pathways/Biological processes	Independent cohort validation
Zhang et al. (2023)	Human peripheral blood (GSE140275,GSE122709,GSE180470)	Bulk RNA-seq	GSE140275: 3 PSD vs. 3 HC; GSE122709: 10 PSD vs. 5 HC; GSE180470: 3 PSD vs. 3 HC	Unspecified	Up: SDHD (FC unspecified), FERMT3 (FC unspecified)	Metabolism-related pathways, hemodynamics	Yes
Ke et al. (2023)	Mouse hippocampus	Bulk RNA-seq	3 PSD vs. 3 Sham	NA	Up: eIF4E, IL-1β, IL-6, TNF-α (FC unspecified) Down: miR-34b-3p (log2FC = −0.98)	Translation initiation, microglial activation, neuroinflammation	No
Qinlin et al. (2022)	Mouse hippocampus	Bulk RNA-seq	8 PSD vs. 8 Sham	NA	Up: FEZ1, NBR1, ULK1, SCOC (FC unspecified) Down: miR-129-5p (FC unspecified)	Autophagy, neuroactive ligand-receptor interaction, calcium signaling pathway, cytokine-cytokine receptor interaction	No
Li et al. (2024)	Mouse hippocampus	Bulk RNA-seq	10 PSD vs. 10 Sham	NA	Up: TLR4, NF-κB, NLRP3, IL-1β, IL-6, TNF-α (FC unspecified)	TLR4/NF-κB/NLRP3 signaling pathway, hippocampal neurogenesis, microglial activation	No

PSD, Post-Stroke Depression; HC, Healthy Control; GEO, Gene Expression Omnibus; MCAO, Middle Cerebral Artery Occlusion; ET-1, Endothelin-1; EA, Electroacupuncture; miRNA, microRNA; DEGs, Differentially Expressed Genes; NA, not accepted; FC, Fold Change. Medication and Specific Drug Class: Drug-free (no recent antidepressant use, typically toxicology-confirmed); Mixed (comprising both medicated and unmedicated subjects); Unspecified (not explicitly reported).

### 5.1 Transcriptomic studies of human peripheral blood

In the context of Post-Stroke Depression (PSD), the peripheral blood transcriptome serves as a critical diagnostic window, bridging acute ischemic injury with systemic affective manifestations. By integrating multiple transcriptomic datasets (GSE140275, GSE122709, and GSE180470), recent analyses have anchored the peripheral PSD phenotype to profound perturbations in energy and substance metabolism (Zhang et al., 2023). Utilizing weighted gene co-expression network analysis (WGCNA) coupled with metabolic gene sets, researchers identified specific metabolic hub genes, notably SDHD and FERMT3, whose expression levels exhibit strict positive correlations with Hamilton Depression Rating Scale (HAMD) depression scores (Zhang et al., 2023). Crucially, diagnostic nomogram models incorporating these two biomarkers demonstrated remarkable predictive efficacy—yielding area under the curve (AUC) values of 0.896 and 0.964—in an independent clinical validation cohort (27 PSD vs. 54 non-PSD cases) (Zhang et al., 2023).

However, applying our critical hierarchy of evidence, it must be noted that the psychotropic medication histories in these retrospective clinical cohorts remain unspecified (Zhang et al., 2023). While these machine-learning-derived biomarkers offer high clinical predictive value, future longitudinal studies must rigorously control for both antidepressant exposure and standard poststroke pharmacological management (e.g., antihypertensives or statins) to definitively establish whether SDHD and FERMT3 represent intrinsic PSD pathology or are partially confounded by complex medical interventions.

### 5.2 Transcriptomic studies of animal brain tissues

At the central level, transcriptomic profiling of rodent focal ischemia models explicitly links post-stroke secondary hippocampal damage to neuroinflammatory and autophagic cascades. Combining brain imaging with RNA-seq, researchers demonstrated that the hippocampus is highly vulnerable to secondary ischemic injury. Within this region, PSD is uniquely characterized by dysregulated non-coding RNA regulatory networks. For instance, in a photothrombosis-induced PSD model, the downregulation of miR-34b-3p and the subsequent upregulation of its target, eIF4E, directly exacerbate microglial activation and the release of pro-inflammatory cytokines (IL-1β, IL-6, and TNF-α); notably, viral-mediated overexpression of miR-34b-3p or silencing of eIF4E significantly alleviates depressive-like behaviors (Ke et al., 2023). Similarly, another whole-transcriptome study utilizing a medial prefrontal cortex vasoconstrictor (endothelin-1, ET-1) model identified massive transcriptomic reprogramming in the hippocampus, primarily enriched in neuroactive ligand-receptor interactions and calcium signaling. This study further delineated a regulatory axis centered around miR-129-5p and core autophagy-related genes (FEZ1, NBR1, ULK1, and SCOC), providing a robust transcriptomic basis for PSD-induced synaptic, inflammatory, and autophagic abnormalities (Qinlin et al., 2022).

Furthermore, longitudinal transcriptomic tracking during interventional research highlights the reversibility of these pathogenic networks. In a PSD model combining middle cerebral artery occlusion with behavioral restriction, a 4-week electroacupuncture intervention at the Zusanli acupoint robustly improved depressive behaviors by reshaping hippocampal neurogenesis (evidenced by increased doublecortin (DCX)-positive newborn neurons and granule cells) and reducing apoptosis in the DG and CA1 areas. Hippocampal RNA-seq confirmed that this therapeutic efficacy is mechanistically contingent upon the transcriptomic downregulation of the TLR4/p38/NF-κB/NLRP3 inflammatory signaling cascades and the systemic regulation of peripheral T and NK cell subsets (Li et al., 2024). Collectively, these findings establish the targeted inhibition of innate neuroinflammation and the restoration of neuroplasticity as core molecular mechanisms for ameliorating PSD.

## 6 Integrative discussion

### 6.1 Shared core mechanisms

**FIGURE 2:**
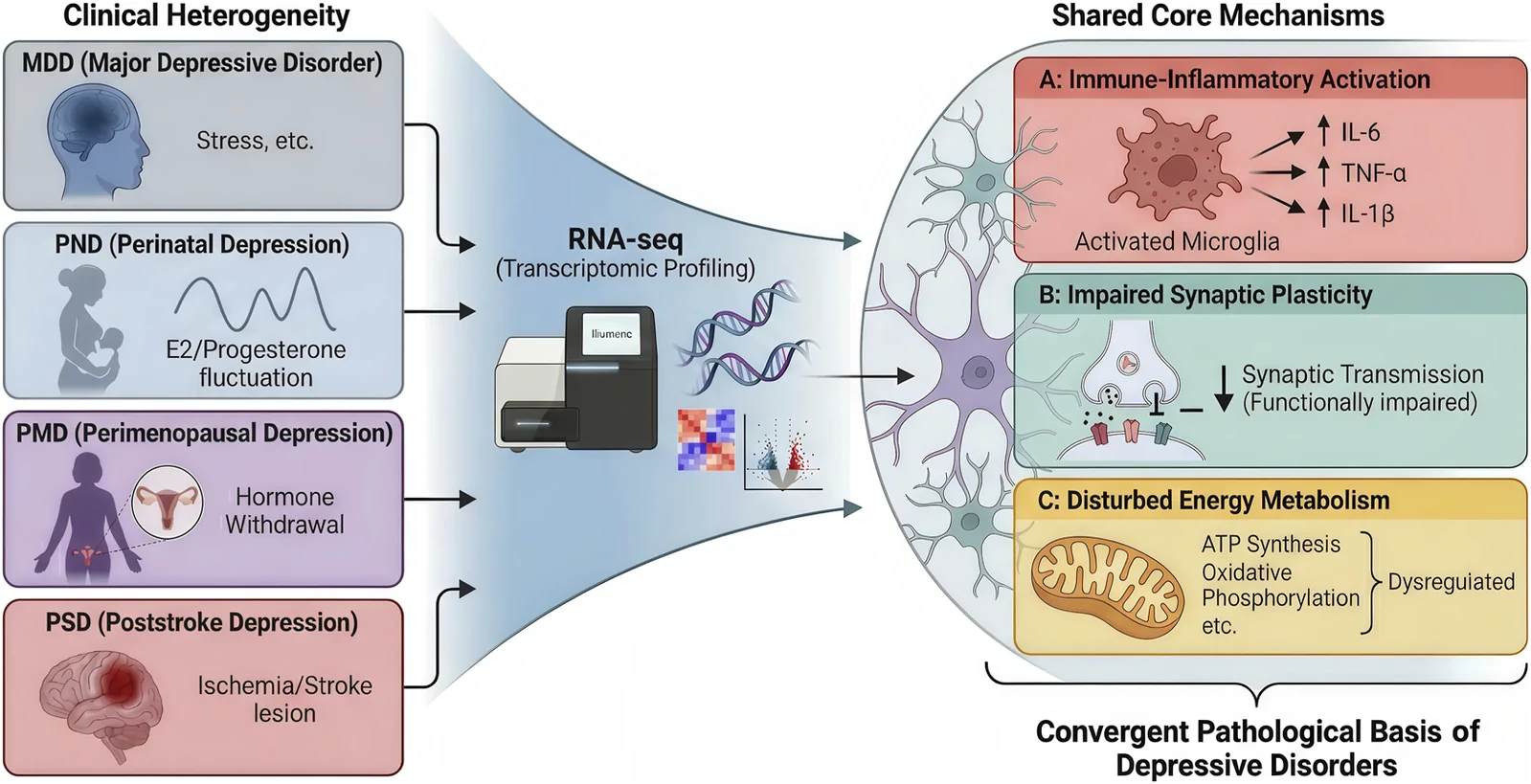
Transcriptomic common mechanisms in depressive disorders.

Despite having different causes, MDD, PND, PMD, and PSD share three main pathological features at the transcriptomic level: immune-inflammatory activation, impaired synaptic plasticity, and energy metabolism dysfunction, as shown in Figure 2. For instance, elevated pro-inflammatory signaling (e.g., IL-6, TNF-α, and NF-κB pathways) is robustly identified not only in the primary baseline of MDD (Yoshino et al., 2021; Chen et al., 2025; Xu et al., 2024), but also as a downstream consequence of hormonal shifts in PMD (Feng et al., 2025) and ischemic injury in PSD (Ke et al., 2023; Li et al., 2024). Similarly, oxidative phosphorylation and ATP synthesis dysregulation appear as universal transcriptomic features across animal models and human peripheral blood in multiple subtypes (Wang Y. et al., 2022; Hofstra et al., 2024; Pan et al., 2018; Zhang et al., 2023). This convergence suggests that despite distinct initiating factors, the pathophysiology of depression involves a generalized transcriptomic disruption, characterized by the maladaptive interplay among inflammation, metabolism, and synaptic pathways. Importantly, these shared transcriptomic signatures provide direct molecular validation for classical etiological models formulated decades ago, formally bridging modern high-throughput data with the foundational macrophage/cytokine theory of depression (Smith, 1991) and the neuroplasticity hypothesis (Duman et al., 1997). Mechanistically, immune-inflammatory activation and bioenergetic deficits within specific glial populations likely disrupt neurotrophic support, driving the structural synaptic impairments in corticolimbic circuits that manifest as core depressive phenotypes. Establishing whether these transcriptomic cascades represent causal, reversible therapeutic targets rather than secondary adaptations remains a critical priority for future targeted perturbation studies.

### 6.2 Etiology-specific transcriptomic signatures

Beyond the shared pathological cascades, transcriptomic profiling delineates etiology-specific molecular signatures that map directly to the unique physiological triggers of each subtype. MDD, serving as the primary baseline, exhibits substantial biological heterogeneity, most notably a profound sexual dimorphism. Specifically, female MDD transcriptomes are preferentially enriched for MAPK pathway alterations, whereas male profiles are dominated by synaptic transmission deficits (Labonté et al., 2017). In contrast, depressions driven by reproductive hormones (PND and PMD) are characterized by a distinct intersection of hormonal fluctuations and immune dysregulation. For example, PND involves specific hormone-inflammation interactions, such as lncRNA Gm14205-mediated inhibition of the oxytocin receptor (OXTR), which triggers astrocytic inflammasome activation (Zhu and Tang, 2020). Similarly, PMD is marked by an imbalance in the inflammation-steroid hormone metabolism network, highlighted by persistent abnormalities in metabolic enzymes like CYP7B1 (Rudzinskas et al., 2021). Conversely, PSD is triggered by acute ischemic brain injury and is transcriptomically defined by the interaction between innate immune responses and ischemia-induced impaired autophagy, involving key genes such as NBR1, ULK1, and SCOC (Qinlin et al., 2022).

### 6.3 Subtype-specific concordance between central and peripheral transcriptomes

Evaluating the concordance between central nervous system (CNS) tissues and peripheral blood is essential for comprehensively synthesizing transcriptomic findings across distinct depression subtypes. While MDD, PND, and PSD possess divergent etiologies, their peripheral transcriptomes robustly and convergently capture systemic immune activation and metabolic reprogramming. For instance, altered bioenergetic pathways are consistently identified in the peripheral blood of both MDD and PND cohorts (Wang Y. et al., 2022; Sforzini et al., 2025; Pan et al., 2018; Mehta et al., 2021), alongside specific metabolic biomarkers, such as SDHD and FERMT3, in PSD (Zhang et al., 2023). However, these peripheral signatures predominantly reflect the systemic inflammatory and metabolic dimensions of the depressive state, rather than direct neural circuit dysfunction. Conversely, cross-subtype comparisons of brain tissue RNA-seq are uniquely positioned to resolve the neuroplasticity dimension, encompassing direct synaptic transmission deficits, microglial activation, and oligodendrocyte dysfunction (Pantazatos et al., 2017; Maitra et al., 2023; Chen et al., 2025). Therefore, while peripheral blood remains an invaluable source for discovering accessible biomarkers across all subtypes, it provides an incomplete proxy for subtype-specific CNS pathology. Future multi-omics study designs should aim to integrate cross-tissue central and peripheral profiling to construct a holistic model of brain-body transcriptomic crosstalk for each distinct depressive etiology.

### 6.4 Cross-species concordance and translational limits of animal models

A dedicated evaluation of animal-to-human concordance is imperative for comprehensively interpreting depression transcriptomics. Systematic cross-species comparisons reveal that direct orthologous gene-level overlap between rodent depression models and human post-mortem tissues remains remarkably low (often <10%). However, integrating these datasets at the network level demonstrates that cross-species concordance manifests robustly within specific biological modules. Systematically, molecular findings related to microglial immune-inflammatory signaling (e.g., TNF-α/IL-6 cascades) and mitochondrial bioenergetic deficits exhibit high cross-species conservation. Conversely, alterations in cortical synaptic transmission and alternative splicing profiles frequently reflect species-specific genomic architectures. Consequently, the appropriate use of animal models lies in interrogating these deeply conserved innate immune and metabolic nodes, while their intrinsic limitation is the inability to phenocopy the complete human cortical transcriptome.

To optimize translational fidelity, animal models must faithfully recapitulate the distinct etiologies of specific clinical subtypes. While chronic unpredictable mild stress (CUMS) serves as a standard paradigm for idiopathic MDD (Willner, 2017), accurately simulating complex endocrine-driven depressions necessitates tailored interventions. For example, deploying a letrozole-CUMS combined model precisely captures the unique neuroendocrine shifts and transcriptomic disruptions inherent to polycystic ovary syndrome (PCOS)-associated depression, which generic stress paradigms fail to induce (Zuo et al., 2023). Similarly, middle cerebral artery occlusion (MCAO) remains the requisite model for post-stroke depression (PSD) (Ke et al., 2023; Li et al., 2024). Ultimately, within these subtype-specific frameworks, targeted genetic and pharmacological perturbations are strictly required to distinguish causal transcriptomic drivers from reactive secondary adaptations, effectively bridging the gap between associative human multi-omics and mechanistic causality.

### 6.5 Transcriptomic reversibility and implications for subtype-specific treatment

Maximizing the translational utility of depression transcriptomics substantially depends on delineating how molecular signatures diverge between treatment responders and non-responders, and evaluating their potential reversibility following effective intervention. Within the datasets synthesized herein, the persistence of shared core pathological nodes—specifically, profound innate immune activation and oxidative phosphorylation dysregulation—provides a plausible molecular substrate for treatment-resistant depression (TRD). Conventional monoaminergic antidepressants, such as SSRIs and SNRIs, primarily target synaptic neurotransmitter levels and often fail to reverse core bioenergetic and neuroinflammatory deficits. Consequently, treatment-resistant populations likely exhibit baseline transcriptomes dominated by these unresolved immunometabolic cascades. Furthermore, prolonged exposure to these agents can induce compensatory immunosuppressive shifts that obscure intrinsic disease signatures. These findings provide a molecular rationale for the variable efficacy of standard antidepressants in heterogeneous cohorts, underscoring the potential of transcriptomic profiling to stratify patients for targeted immunometabolic interventions rather than empirical SSRI/SNRI trials.

Furthermore, interpreting these transcriptomic divergences underscores the critical importance of subtype-specific therapeutic paradigms. The etiology-specific signatures unique to post-stroke depression (PSD) detailed in our synthesis—such as the dysregulation of SDHD and FERMT3 linked to acute ischemic injury—indicate that standard MDD pharmacotherapies may be mechanistically misaligned for these patients (Zhang et al., 2023). Achieving robust transcriptomic reversal in PSD is expected to require interventions capable of mitigating ischemia-induced neuroinflammation and promoting post-stroke neurovascular repair. Similarly, the hormone-inflammation network imbalances defining PMD (e.g., persistent CYP7B1 anomalies) (Rudzinskas et al., 2021) and PND (e.g., Gm14205-mediated OXTR inhibition) (Zhu and Tang, 2020) suggest that effectively reversing these states would benefit from targeted neuroendocrine-immune modulation or multidimensional somatic neuromodulation, rather than relying solely on generic, monoamine-centric approaches.

Despite medication status being predominantly regressed as a covariate in the current literature, future longitudinal multi-omics investigations should increasingly prioritize treatment-stratified analyses. By explicitly evaluating how the subtype-specific transcripts identified herein respond to distinct therapeutic modalities, the field can further validate these molecular signatures as predictive biomarkers. Ultimately, delineating the reversibility of these pathogenic networks will facilitate the transition from empirical prescribing to precision neurotherapeutics, aiming to ensure that clinical interventions are mechanistically calibrated to the distinct transcriptomic etiologies of MDD, PND, PMD, and PSD.

### 6.6 Multidimensional transcriptomic profiling beyond conventional DEGs

Recent peripheral blood transcriptomic studies have increasingly moved beyond conventional differential gene expression (DEG) analyses, adopting higher-order systems biology frameworks to parse the clinical heterogeneity of depressive disorders. Approaches such as Weighted Gene Co-expression Network Analysis (WGCNA) and molecular biotype stratification allow for the identification of functionally coordinated gene modules. For example, Sforzini et al., 2025 stratified MDD patients based on serum C-reactive protein levels, revealing immunometabolic dysregulation specific to an inflamed biotype. Employing WGCNA, Wang M. et al. (2025) identified an RNA splicing module tightly associated with suicidal ideation, demonstrating the utility of network analysis in mapping complex clinical phenotypes to specific transcriptomic clusters. Furthermore, molecular subtyping by Sirés et al. (2025) confirmed that treatment-resistant depression (TRD) forms a distinct biological architecture characterized by widespread epigenetic and vesicle metabolism alterations, diverging from classical monoaminergic deficits.

This multidimensional network perspective extends to dynamic clinical prediction across various depression subtypes. Overcoming the limitations of cross-sectional designs, longitudinal transcriptomic tracking provides prospective biomarkers for disease onset. In Perinatal Depression (PND), Mehta et al. (2021) and Björvang et al. (2025) demonstrated that specific immune-inflammatory shifts detected as early as the third trimester function as early-warning sensors, effectively predicting the trajectory of postpartum depressive episodes. In Poststroke Depression (PSD), Zhang et al. (2023) integrated WGCNA with machine learning algorithms to isolate core immune and metabolic hub genes, yielding a diagnostic model with high predictive efficacy following ischemic events.

Furthermore, the exploration of post-transcriptional modifications and non-coding RNAs is crucial for comprehensively capturing the complex transcriptional profile of major depressive disorder (MDD), especially in readily available peripheral alternative samples. For instance, alternative splicing and intron retention (IR) offer a unique analytic layer beyond total gene expression. A recent study by Okada et al. demonstrated that mapping IR changes in human peripheral blood mononuclear cells (PBMCs) captures subtle yet critical alterations intertwined with innate immunity and ciliary assembly (e.g., STAT1, AHI1, and CEP104) (Okada et al., 2024). Another study reanalyzed a whole-blood RNA-seq dataset from ketamine-treated TRD patients by employing intron retention (IR) metrics (Okada et al., 2025). The findings revealed that ketamine non-responders exhibited an elevated baseline inflammatory and viral infection signature. Crucially, specific IR genes associated with these pathways were restored following ketamine treatment irrespective of the clinical response status, suggesting that non-response may stem from an overwhelming baseline inflammatory load rather than an absolute lack of pharmacological action (Okada et al., 2025). This IR profiling provides a highly sensitive, post-transcriptional method for evaluating rapid antidepressant responses, capturing predictive molecular layers that standard gene-level counts inherently omit. Similarly, the non-coding transcriptome presents highly stable biomarker candidates. Zhang et al. characterized circular RNA (circRNA) profiles in the peripheral blood of drug-free MDD patients, identifying a specific diagnostic panel of upregulated circRNAs (e.g., hsa_circ_0002473 and hsa_circ_0079651) that are deeply integrated into neuroplasticity-related ceRNA networks (Zhang et al., 2022). Furthermore, exploring these advanced transcriptomic layers is pivotal for precision psychiatry. Utilizing whole-blood RNA-seq, Cathomas et al. successfully delineated molecular signatures associated with rapid antidepressant responses to ketamine in treatment-resistant depression (TRD). Notably, baseline expression of specific glutamate signaling genes (GRM2 and GRIN2D) successfully distinguished ketamine responders from non-responders prior to treatment (Cathomas et al., 2022). Collectively, these anchor studies illustrate that transitioning from standard DEG analysis to multi-layered transcriptomic profiling significantly enhances blood-based molecular stratification and therapeutic prediction (detailed cohort characteristics and analytic layers are summarized in Supplementary Table S1).

## 7 Conclusion and future perspectives

### 7.1 Current methodological and translational challenges

The translation of transcriptomic findings into clinical psychiatric practice is fundamentally constrained by methodological barriers. Post-mortem brain studies, the primary source of central nervous system data, are inherently confounded by lifelong pharmacological exposure and medical comorbidities. This vulnerability is especially critical for MDD. As foundational cross-disorder studies demonstrate, MDD exhibits arguably the most subtle transcriptomic signatures among major psychiatric conditions, making its molecular profile uniquely susceptible to experimental noise (Gandal et al., 2018). This inherent vulnerability is further compounded by the highly inconsistent reporting of critical pre-analytical variables—specifically RNA integrity number (RIN), post-mortem interval (PMI), and sequencing depth—across the primary literature. While these technical parameters actively drive transcriptomic variance, many studies fail to provide sample-level data, relying instead on statistical covariates to regress these effects *post hoc*. However, mathematical regression cannot biologically reconstruct specific transcript biotypes that have undergone physical post-mortem degradation. This overreliance on statistical adjustment, coupled with disparate reporting standards, makes it virtually impossible to separate true biological signals from pre-analytical artifacts. Consequently, differentially expressed genes (DEGs) identified in cohorts lacking stringent pre-analytical cutoffs demand rigorous methodological skepticism.

Furthermore, technical divergence across studies compromises cross-cohort reproducibility. The historical reliance on bulk RNA-seq, while effective for delineating macroscopic pathway alterations, fundamentally conflates genuine intracellular transcriptional shifts (cell-state effects) with stoichiometric variations in resident cell populations (cell-composition effects) (Wang and Dwivedi, 2025). This analytical limitation, exacerbated by the frequent absence of sex-stratified analyses and the systemic under-investigation of complex, etiology-specific subtypes (e.g., PND, PMD, and PSD), significantly restricts the mechanistic interpretation and generalizability of existing datasets.

### 7.2 Strategic avenues for future research

To transcend these limitations and propel the field toward precision psychiatry, future investigations should prioritize several strategic paradigms. First, to disentangle the profound cellular complexity of the central nervous system, future multi-omics designs must increasingly integrate single-cell/single-nucleus transcriptomics (sc/snRNA-seq) with spatial profiling. This high-resolution approach will facilitate the precise mapping of dysregulated corticolimbic circuits and the elucidation of cell-type-specific pathogenic cascades, identifying precise loci of injury (Labonté et al., 2017; Smajić et al., 2022; Reiner et al., 2020; Ruzicka et al., 2020; Velmeshev et al., 2019; Li and Wang, 2021; Salmén et al., 2018).

Second, given the pronounced sexual dimorphism characterizing depression transcriptomes, pooling mixed-gender samples risks obscuring critical biological signals and generating artifactual data. Future study designs must establish sex-stratified analysis as a mandatory baseline standard. Integrating these dimorphic baselines with models of PND and PMD will be crucial for understanding hormone-driven depressions not as isolated entities, but as context-dependent exacerbations of underlying transcriptomic vulnerabilities.

Third, because transcriptomic data inherently reflects reactive gene expression fluctuations, differentiating causal drivers from secondary adaptations requires orthogonal validation. Integrating transcriptomics with genome-wide association studies (GWAS), epigenomics, and proteomics will enable the construction of robust causal networks. Concurrently, systematically evaluating cross-species concordance and applying targeted perturbations in subtype-specific animal models will be essential to validate these networks as viable therapeutic targets.

Finally, the field must pivot from an exclusive focus on idiopathic MDD toward a broader inclusion of discrete clinical entities, including peripartum, perimenopausal, and post-stroke depressions. Crucially, transitioning from cross-sectional baseline profiling to longitudinal, treatment-stratified designs will allow researchers to evaluate transcriptomic reversibility, thereby identifying predictive biomarkers for treatment response and resistance.

### 7.3 Concluding remarks

In conclusion, RNA-seq technologies are systematically deconstructing the highly heterogeneous syndrome of depression into biologically resolvable transcriptomic modules. The evidence synthesized herein indicates that while MDD, PND, PMD, and PSD are precipitated by divergent physiological triggers, their molecular etiologies converge upon shared networks of immune-inflammatory activation, synaptic impairment, and metabolic dysfunction. As advanced spatial-transcriptomic modalities mature and subtype-specific cohorts expand, the continued mapping of these pathogenic networks holds the promise of guiding psychiatric practice away from empirical, symptom-based management toward mechanistically calibrated precision neurotherapeutics.
