# Supplementary material for: Progress and challenges in exploring the pathogenesis of depressive disorders via RNA sequencing
Source: Front Genet. 2026 Aug 28;17:1792849. doi: 10.3389/fgene.2026.1792849 (PMC13563219; doi:10.3389/fgene.2026.1792849)
Supplement: Supplementary file 1 [file Table1.docx]

**Table S1. Summary of Multi-layered Peripheral RNA-seq Studies Beyond Conventional DEGs**

| **Author (Year) [Ref]** | **Sample Source** | **Cohort Characteristics** | **Analytic Layer** | **Main Findings** | **Relevance to Prediction / Stratification** |
| --- | --- | --- | --- | --- | --- |
| Naoto Okada (2024) [76] | PBMCs | Size: 8 MDD vs 6 HC Medication: Drug-free (>4 weeks washout) | Intron Retention (IR) | Discovered significant IR variations in innate immunity and cilia-related genes (e.g., STAT1, AHI1) | Yields robust diagnostic markers that act as physiological stress sensors, extending beyond standard DEGs. |
| Okada et al. (2025) [77] | Whole blood | Size: 26 TRD vs 21 HC  Medication: Drug-free(>4 weeks washout) | Intron Retention (IR) | Non-responders were characterized by a baseline state of significantly elevated viral-like inflammation. Ketamine restored specific innate immunity-related IR genes (e.g., EEF1A1, NLRC5) in both groups, but was insufficient to overcome the severe inflammatory overload in non-responders | Establishes IR as a robust physiological sensor to distinguish ketamine responders from non-responders, defining treatment resistance as a threshold effect of inflammatory overload rather than constitutional insensitivity. |
| Donghui Zhang (2022) [78] | Peripheral blood | Size: 29 MDD vs 19HC Medication: Drug-free (>3 months washout) | circRNAs | Identified a highly specific panel of upregulated circRNAs (e.g., hsa_circ_0002473, hsa_circ_0137187) | Provides stable, testable non-coding biomarker candidates linked to neuroplasticity networks. |
| Flurin Cathomas (2022) [79] | Whole blood | Size: 26 TRD vs 21 HC Medication: Drug-free (>2 weeks washout) | DEGs (focus on treatment response) | Baseline expression of GRM2 and GRIN2D was significantly upregulated in ketamine responders | Predicts rapid antidepressant response, enabling precision psychiatry stratification for TRD. |
